# I move, therefore I am. Meaning, philosophy and physical literacy

**DOI:** 10.3389/fspor.2026.1920693

**Published:** 2026-10-01

**Authors:** Jens Erling Birch, Øystein Winje

**Affiliations:** 1 Department of Primary and Secondary Teacher Education, Oslo Metropolitan University, Oslo, Norway; 2 Department of Sport Science and Physical Education, Universitetet i Agder, Kristiansand, Norway

**Keywords:** existentialism, lifespan, meaning, movement, Nietzsche, physical literacy

## Abstract

Physical literacy (PL) is not simply being physically active. PL can be described as the motivation, confidence, physical competence, knowledge and understanding to value and take responsibility for engagement in lifelong physical activity. We will inquire into the philosophical underpinnings of the existentialist component of *why* one should value and take responsibility for engagement in lifelong physical activity. We discuss this “why” question by elaborating on a philosophical and existentialistic interpretation of *meaning.* Our belief is that by giving meaning to our engagement in physical activities we can maintain motivation, confidence, competence, knowledge, and understanding during our lifespan. After a presentation of some initial elements of existentialism, PL and a discussion of meaning in a philosophical sense, we will continue by discussing why it is important to find meaning in physical activity, and then what constitutes a meaningful life. We argue that the quest for philosophical meaning arises from the vulnerability of human life and the meaninglessness of a material universe. We propose to offer one type of answer via Lucretius’ thoughts on death, Owen Flanagan and Thomas Nagel's reflections on developing a meaningful lifestory, and then present Friedrich Nietzsche's idea of the so-called *eternal recurrence*. From there, we present our interpretation of the eternal recurrence as a question one must ask oneself when confronted with questions of meaning and what one should do with one's life. In the light of Nietzsche, we attempt to discuss physical activity and the holistic idea of physical literacy as an important part of living, of human existence and one possible way of finding meaning throughout our lifespan. Finally, our inquiry will lead us to a picture that presents physical literacy not only as something important, but first and foremost as something *vital*.

## Introduction

Physical literacy is not simply being physically active. In line with Margaret Whitehead's (1) holistic characterization, physical literacy can be described as the motivation, confidence, physical competence, knowledge and understanding to value and take responsibility for engagement in lifelong physical activity (2). In this manuscript, we inquire into the philosophical and existentialist component of *why* one should value and take responsibility for engagement in lifelong physical activity. As Whitehead (3, p. 129) highlights, the concept of physical literacy is grounded in *both* phenomenological and existential philosophy. Although scholarships on physical literacy frequently foreground phenomenology (4), existentialism has tended to receive comparatively less explicit attention within this literature [Durden-Myers (5) is a recent exception]. In accordance with Whitehead's philosophical fundament and contributions by Durden-Myers et al. (6), we discuss the abovementioned “why” question by elaborating on a philosophical and existentialistic interpretation of *meaning.* Our belief is that by giving meaning to our engagement in physical activities, we can better maintain motivation, confidence, competence, knowledge, and understanding during our lifespan.

Our inquiry proceeds in several steps. First, we present some initial elements of existentialism in general, and then more specifically look at existentialism and PL, followed by a discussion of what we talk about when talking about meaning in a philosophical and existentialistic sense. Third, we continue by discussing why it is important to find meaning in physical activity. Then, using Owen Flanagan's (7, 8) work on what constitutes a meaningful life, our departure will be that the quest for philosophical meaning arises from the vulnerability of human life and the meaninglessness of a material universe made up of particles and forces. Such a universe is (arguably) the vantage point from an anti-Cartisian monism (see 9). Fifth, we attempt to offer one type of answer (or a question) to our individual quest for meaning in such a universe. We will do this via Lucretius’ thoughts on death, Owen Flanagan and Thomas Nagel's reflections on developing a meaningful lifestory, and then present Friedrich Nietzsche's idea of the so-called *eternal recurrence*, partly anticipating Jean-Paul Satre's later existentialism (10). From there, we will display our interpretation of the eternal recurrence as an existential hypothesis: a question one must ask oneself when confronted with questions of meaning and what one should do with one's life. In the light of Nietzsche's early existentialism, we intend to discuss physical activity and the holistic idea of physical literacy as an important part of living, of human existence and one possible way of finding meaning throughout our lifespan. Finally, our inquiry will lead us to a picture that presents physical literacy not only as something important in an instrumental sense by increasing our medical-biological health, but first and foremost as something of existential importance, helping to build and express identity and self-understanding.

### Existentialism and PL

The philosophical underpinnings of PL share an oppositional stance towards Cartesian dualism. From a substance physical monism (9), PL is anchored in *embodiment* (1). Embodiment is standardly accustomed to the relational and holistic aspect of human-world interaction, and hence phenomenology (11). Although existentialism, alongside monism and phenomenology, is one of the three philosophical pillars of PL it has to a lesser extent received its share of attentional limelight. When we talk about games, sports and physical activity, existential philosophy is considered an important aspect of what gives an activity its meaning. We will return to how this literature understands meaning below, and how the question we pursue differs from it in the next sections.

Whether an action, and hence any kind of movement is considered an important part of human life, is an existential question. Our framework is loosely built upon such a question. From a monistic standpoint then, it is also our starting point that human life is rather short and vulnerable. We might even say that these limitations are embodied and embedded in our life on earth (see 12). They are, so to speak, existential building blocks from where we try to develop a sense of self (13). From physical monism it also follows that it is from the human body that we must develop this sense of self. We will therefore start with a brief description of some overlapping ideas that form, if not an existentialistic framework, at least some shared existentialistic ideas. From there, we describe in more detail the sense of meaning and human anthropology that we try to bring to discussions on PL, philosophy, and meaning.

It seems uncontroversial to claim that existentialism is more of a philosophical movement than a distinct school of thought (14). Still, names like Heidegger, Sartre, Marcel and Merleau-Ponty are standardly regarded as existentialist thinkers occupied with human existence rather than analytic epistemology or philosophy of language. Arendt (15, p. 173) traces the existential tradition to Kierkegaard in the mid 18 hundreds, followed by Nietzsche 30–40 years later. Kierkegaard and Nietzsche direct their philosophical lens away from a focus on rationality and objectivity, and towards awareness of death, anxiety, nihilism, subjectivity, freedom and personal choice. These central themes influenced Heidegger, Jaspers and Sartre so much so that existentialism as a term appeared a century later (16). Baert (17) describes “the existentialist moment” as a movement that evolved in the wake of World War II, re-emerging the conceptual framework of Kierkegaard and Nietzsche on the human condition, anxiety, death, freedom and meaning(lessness). According to Aho (18) it is hard to find a unifying theory in existentialism. Instead, there are some overarching and conjoining ideas. Most central to our inquiry are:

A) *Nihilism*: Nietzsche (19, GS 125) saw the transition from a pre-modern religious worldview to a secular and scientific (perhaps only of physical substances) one as leaving humans without a moral framework. A lack of external morality leaves us in modernity's nihilistic abyss where anxiety, alienation, and meaninglessness rules.
B) *Existence Precedes Essence*: Sartre's (20) famous statement means our essence is neither determined nor more important than how we create ourselves and our path through choices and actions.
C) *Freedom*: Humans are free and responsible for who we are and what we do. This freedom, however, is not unconditioned. Sartre's own account distinguishes freedom from facticity: we are free, but always from within a situation we did not choose, a body, a history, a set of material circumstances (21). A century before Sartre, Kierkegaard (22) described anxiety as an inherent part of human freedom. Existential anxiety may be described as an awareness of uncertainty, freedom, and the potential meaninglessness of life. Yet Kierkegaard also suggested that commitment and action provide pathways for navigating such a condition. This also means we carry a burden on our shoulders that we cannot escape (see e.g., 19, 21, GS 341).

This article may be read without commitment to existential philosophy *per se*. Still, central themes like those described above are certainly relevant. Therefore, we are more interested in the themes than the -ism(s). For example, Heidegger (23) highlighted how human existence is shaped by an awareness of mortality. As we have seen, existentialist philosophy also highlights that individuals bear responsibility for creating meaning in their own lives. The former is a central pillar to our initial philosophical discussion, and the latter is our focus on the interplay between meaning and PL. It is worth being explicit, from the outset, about the philosophical traditions this article rests upon. Kierkegaard, Nietzsche and Sartre, belong to a European existentialist tradition, which focuses on the individual. Other traditions begin elsewhere. Andean concepts of *buen vivir*, for instance, locate the good life in community and nature rather than starting with individual choice. Gudynas (24) cautions against transposing the concept to other settings. We attempt to offer *one* contribution to a hopefully ongoing discussion; a comparative treatment lies beyond our scope.

Some of the themes described above have already been addressed by writers interested in physical activity (25), others have a stronger connection to sports than PL. Aggerholm (26) for instance, draws on Kierkegaard to show how elite athletes can continuously find meaning. Inglis (27) argues that sport can fill an existential void and is more freely chosen than work because sport may be seen as unnecessary. This literature exemplifies how human existence is a subjective meaning-making in a world we are thrown into and therefore must define values and purpose to live authentic lives.

Durden-Myers (5) lists Kierkegaard, Heidegger, Sartre and Merleau-Ponty as some of the prominent figures of European existentialism underpinning PL. Durden-Myers highlights individual freedom, subjectivity, responsibility, personal choice and meaning amongst influential existentialistic themes. In a similar way Durden-Myers reads Sartre's “existence precedes essence” into PL: We are not born with a fixed purpose but create ourselves through engagement and choice. From such a background, we could say human existence is signified not by an ontology of *being* then, but by an existence of *becoming* (28, TI, The Four Great Errors), or a continuous striving (22, p. 90; see also 25). To look at existence as something becoming, is reflected in the physical literacy *journey* (29) – a continuous project of *becoming who you are* (19, GS 270, GS 335) throughout life.

In line with Durden-Myers, we agree that existentialism provides a lens through which physical literacy can be comprehended as a component of authentic human existence. As such, PL and existentialism are deeply intertwined. Inspired by this relationship, we aim to dwell deeper into meaning in a philosophical sense below. Regarding meaning and PL, Durden-Myers (5) follows in the footsteps of Aggerholm (25, 26), Standal and Moe (30) and Durden-Myers et al. (13). In this literature, something, like a game, becomes meaningful through its context and subject-world relations (25, 26), and motor intentionality (a Merleau-Ponty concept) appears in the meaning of movement patterns (30). Durden-Myers (5) likewise describes meaning as making sense of a situation, a game, a pattern of movement. From this point of view, meaning is a relationship between an intentional movement and the environment. This is not the sense of meaning we will pursue. Where this literature asks how a given movement or game becomes meaningful, we ask the prior question: how and why can/should movement be part of a meaningful life *at all*? From our perspective, meaning is how we cope with our existence given that our essence is physical. It is both what we are doomed to, to paraphrase Sartre (21), and the responsibility we are left with if there are no external values or goals. Physical monism also leaves us with a certain body, and thus certain ways to move and express who we are with, through being/becoming such a body. We continue with a discussion on meaning in the next section. Our interest lies in how humans, through PL, can actively exercise freedom and authenticity by choosing how they move in ways that resonate with who they are and who they may become and thus ultimately find meaning throughout the lifespan.

### Philosophy and different interpretations of meaning

A first and central task is distinguishing between at least two overarching interpretations when thematizing meaning: instrumental meaning and existential meaning. A closely related distinction has long been drawn in philosophy: Robert Nozick (31) separates the extrinsic value something has as a means to a further end from the intrinsic value it has in itself, and argues that meaning, though it always points beyond itself, must finally rest on something intrinsically valuable rather than on something merely instrumental (31, p. 610). Our distinction between instrumental and existential meaning follows the same logic. When adopting an instrumental perspective on physical literacy across the lifespan, the primary argument concerns the rational, the utility, or the beneficial outcomes associated with physical activity. Here, physical activity is meaningful because it produces an advantage, result, or benefit external to the activity itself. From an evolutionary viewpoint, for example, one might argue that children engage in play to develop motor competencies to interact more effectively with their environment. From a health-oriented perspective, adults may continue to engage in physical activity by going to the gym because doing so promotes health, prevents illness, and reduces the risk of injury. Such an interpretation of meaning frames physical activity and subsequently physical literacy as means to an end.

Although these are certainly good outcomes, this article does not seek to foreground such instrumental interpretations of meaning. Instead, our focus is the existential meaning of physical literacy, one of the things we believe distinguishes mere physical *activity* from physical *literacy*. An initial question may highlight this difference: Why are some of us so obsessed in a physical activity that it not only transcends the obvious instrumental benefits such as improved health but may cause harm, injury and serious risks including death? Our contention is that instrumental meaning is rarely sufficient to sustain long-term engagement and scarcely explain the urge to do extensively more than health recommendations. Motivation tends to decrease when activity is justified solely by external outcomes. In contrast, physical activity becomes resiliently embedded in a person's life when its significance is experienced as existential, when the activity is tied to identity, self-understanding, and the ongoing project of leading a specific way of life.

Examining motivation, confidence, physical competence, knowledge and understanding through the lens of existential meaning highlights the intrinsic and identity-shaping dimensions of how physical activity may *become* physical literacy. *Motivation* becomes inherent rather than instrumental, emerging from the meaningfulness of the activity itself. *Confidence* does not solely come from some external good produced by an activity, but because activity is integrated into one's life as something existentially significant. Willingly choosing to become physically active is also to willingly refuse something else. When a choice is made due to existential rather than instrumental reasons, we might experience confidence in choosing what seems right, perhaps even good. Valuing an activity as something important in this sense can also motivate the desire to develop one's *physical competence*, *knowledge,* and *understanding* of the activity itself and engagement in it. The type of existential commitment we are addressing here may hopefully guide one's reasons for choosing to continue a physical literacy journey rather than departing from a trail which sometimes will be strenuous. Many of us have experienced the thrill of starting with a new physical activity, and then, when the beginners’ initial development of physical competence related to the activity plateaus, our motivation and confidence suffer, and instead of staying on the strenuous path, we switch to a different path, another activity. The activities that stay with us through a lifetime are often the self-chosen and self-driven ones, the activities in which existential rather than instrumental meaning is at stake. When instrumental meaning diminishes and existential meaning has not yet taken root, we may tend to give up the activity, or switch to another. Whether one stays with a single activity or moves between several is, by itself, less decisive than whether the choice is a reflected one. Both paths can sustain a lifelong engagement, but it surely helps if we become aware of *why* we continue, give up, or begin anew. The point we wish to emphasize is that strengthening the existential meaning of physical activity can make instrumental justifications less significant. When an activity is valued for its own sake, rather than for the benefits it produces, it becomes more deeply rooted, more personally significant, and ultimately more likely to remain part of a life, however that life is lived, and whatever one's movement (in)capabilities are. This should not be read as a case for activity as such. Provided stillness is chosen rather than merely defaulted into, then a life with long stretches of inactivity, rest or contemplation may provide meaning just as fully as a life of movement. Physical literacy also includes knowing when not to move.

### The world and human existence

After the section on what we talk about when we talk about meaning above, we will now continue our inquiry by presenting a view of meaning as *meaning of life*. The philosopher Owen Flanagan refers to the search for an answer to what makes life worth living as *The Really Hard Problem* (7, 8). Flanagan initially claims that excessive rumination on questions concerning the meaning and purpose of life may be a waste of time and, in the worst case, increase the likelihood of perceiving existence as unbearable. Human life may in fact be simpler and more tolerable for those who do not reflect on such questions. But suppose that people interested in gaining knowledge (in general) engage in such reflection—what then?

A possible starting point could be to discuss whether the pursuit of happiness provides meaning in life. Flanagan argues that happiness is insufficient to guarantee that life is worth living, since a life may contain a great deal of happiness and still be meaningless: some individuals appear to derive happiness from committing sadistic acts, yet we would hardly attribute meaningful lives to them. It is equally reasonable to claim that a life may be full of meaning and yet be unhappy: self-sacrifice for the sake of others may be meaningful even if it results in personal misfortune. In relation to physical literacy, one might argue that a rather substantial amount of time spent on physical activity involves effort, struggle, discomfort, and even pain. If you want to improve your running stamina or become a stronger climber, you must be motivated and confident enough to push the limits of your own capability, which mainly involves struggle and (temporary?) unhappiness. This does not imply that happiness is unimportant or incapable of contributing to a meaningful life. Flanagan maintains that just as important as joy and happiness is the fact that human beings, across cultures and throughout history, have found value in developing an identity and expressing it, particularly in personal relationships.

What kinds of self-expression and identity can be said to be secure, positive, valuable, and thereby capable of establishing meaning in a life? This is a question tied to one's worldview and human anthropology - that is, to what the world and human beings are. According to Flanagan, answering this question rests on two fundamental extremes:

1) A deity has created the universe, and the beings within it are divine. Identity, self-worth, and meaning are therefore grounded in the divine.
2) Beyond knowledge of the Big Bang and subsequent cosmic and biological evolution, we do not know why anything exists at all. Such a scientific explanation (or lack thereof) easily leads to questions about what significance or meaning anything has, including oneself.

Flanagan argues that the religious approach does not necessarily provide a better answer to the question of life's meaning. Beyond religious doubt Flanagan claims external sources of meaning (such as gods) are problematic because the divine plan is difficult to grasp, and because it is unclear whether the deity or deities are genuinely good. If we ruminate long enough, religion may offer no more consolation than science. In short, both posits something external to my own inner sense of meaning – and that is unsatisfactory. We should therefore look elsewhere to establish meaning in our lives. Friedrich Nietzsche, to whom we shall return, also believed that the extremes outlined above lead to what he calls meaninglessness and nihilism. Naturalistic and scientific explanations do not align with theological values, and science itself has in many ways rejected teleological values (things do not strive toward a purpose). To avoid falling into a nihilistic vacuum, human beings must seek values and meaning elsewhere than in religion or science. We must become our own authorities and construct our identity and meaning from other foundations. In a sense, this involves seeking meaning in our own lives - a form of existentialism. According to Flanagan, we can, from birth to death, establish and later leave behind meaningful traces, a lifestory. We develop such a lifestory in situations and relationships where we express our identity, hopefully in positive ways. He calls this a *naturalistic transcendence*. We will argue here that this is where meaning regarding PL is found: In the choices and reasons we make about why, how and which physical activities to pursue. These choices are how we express, through movement, the identity Flanagan places at the center of a meaningful life.

In this manuscript, we will (reluctantly) departure from a scientific understanding of the world. This is in part because we believe that physical literacy has a relentless commitment against substance dualism and towards (physical) monism. A commitment towards (physical) monism should also lead to a commitment towards contemporary science (see 9). Why is a scientific world view unsatisfactory for establishing meaning in our lives? Ontological physical monism claims there exists nothing beyond the physical particles of which we and the universe are composed. Physical particles, such as atoms, do not possess goals or purpose, science tells us. If everything that exists consists of physical particles devoid of inherent meaning, does this imply that neither the universe, the Earth, nor human beings possess any significance whatsoever - that our lives are entirely meaningless? A commitment against Cartesian substance dualism, and towards (physical) monism will eventually face this problem (11). Within such a scientific and/or naturalistic worldview, life does not justify itself. We agree with Flanagan that what makes the search for meaning so difficult is the acknowledgement that there appears to be no meaning or direction in the universe, and consequently none in our world or in our lives. The question of meaning, in this sense then, is above all tied to an ontological viewpoint. We do not agree with Flanagan though that ontological physical monism would dismiss any kind of existentialism. Flanagan seems to argue that accepting that everything is physical is also to posit meaning externally (contrary to existentialism), but if everything is physical then everything about us humans, also our very existence and any way we act within it, must also be physical. This cannot be seen as something external. Rather, an ontological commitment leads secondly to an existential consequence. In a physical universe, this consequence follows from the vulnerability of humans. If we are nothing more than physical bodies, then this vulnerability is constitutive. It confronts us with our own fragility, and at the same time makes physical literacy matter existentially, not merely instrumental and external. Physical monism and existentialism are complimentary by displaying a perspective where most of us will live on planet Earth for less than a hundred years and during this lifetime all things we hold dear might be taken from us in an instant. We might say that our human existence embodies a constant search for meaning because neither our species nor our individual lives seem to have any meaning in and by themselves. What we do during our rather short and limited lifetime on earth then, may be understood either as completely meaningless *or* if it is *only* this short lifespan: of utmost importance.

From the perspective of PL, the fragility of human life prompts the importance of making conscious decisions that shape a lifestory expressing our identity. If this also involves other people and leaves positive traces; we should not complain. This does not simply lead down the path of instrumental meaning because, as argued above, instrumental meaning is rarely enough to make us continue our physical literacy journey. It is the kaleidoscopic interaction between our motivation, confidence, understanding, knowledge, and physical competence that keeps us engaged. In other words, we need to use this kaleidoscope to identify physical activities that engage us and provide existential meaning. We will return to the latter rendition after a brief philosophical diversion on Lucretius, Nagel and death - leading up to Nietzsche.

### Death is nothing to us. Or is it?

Like Flanagan, the Roman philosopher Lucretius (95–55 BCE) was a type of naturalist (or atomist). He pointed out a peculiar phenomenon: human beings seem to fear death, yet no one appears to fear the time before we are born. In *De Rerum Natura* (On the nature of things) Book III, Lucretius (32) makes a case for why we shouldn't fear death. Lucretius’ atomism amounts to the perspective that everything in the universe is made of atoms. This also holds for humans – there is no immaterial soul in Lucretius’ world view. Counterintuitively, this is one of the reasons, as we shall see, that we need not fear death, or in other words: death is nothing to us. The logic of the argument is straight forward: Because I am made of atoms, then I did not exist before birth. Death is the end of my existence, and hence after death I do not exist. To me then, time before birth is identical to time after death. Since I do not fear the time before death, I should not fear time after death – death is nothing to me, it is simply void, empty, *nihilo*. Death offers neither pleasure nor pain - just like before birth. In Lucretius’ view then, what we often call the soul is ultimately nothing distinct from the body and this should comfort us.

While we can appreciate the logic of Lucretius’ argument, it is not very comforting. More importantly, Lucretius’ point is that death is a part of the nature of things, so it will come sooner or later. Instead of worrying about this inevitable and uncontrollable event, we should focus on living our lives to the fullest. In Book II of *De Rerum*, Lucretius sums up his Epicurean stance toward such a life: to avoid bodily pain, fear and anxiety, and to better enjoy the pleasures of the senses. It is the fear of missing out, not having enough time with our loved ones, or completing some long-standing project that terrifies us. We fear life, in the same way that we fear death. But just as we cannot control death, we cannot control the future. So instead of worrying about past or future life, we must accept our one and only life and appreciate its preciousness. Although we can agree with Lucretius that we should appreciate the time we have on earth to a larger extent, this insight does not automatically solve our existential dreads.

American philosopher Thomas Nagel (33) highlights the obvious insight that death is what deprives us of life. If death did not occur, we would continue to live. What we fear, according to Nagel, is not the time after death has occurred, but that death deprives us of opportunities—even good opportunities. Human vulnerability lies in the recognition that, sooner or later, all opportunities will be gone. Beyond this existential vulnerability, we are also vulnerable in the sense that even minor accidents, arising purely by chance or misfortune, can rob us of substantial opportunities. A sudden injury to your knee while playing football, with subsequent operations and rehabilitation, might leave you unable to return to future play. Your ability to participate in activities that were once meaningful might now be permanently blocked, forcing you to make significant changes and choices regarding your own physical literacy journey. A central aspect is thus how adaptable you are. Do you have the motivation, confidence, understanding, knowledge and physical competence to find a new activity or to adapt to your new (embodied) reality and find ways to participate even after an injury? We are in many ways both fragile and pitiable, so the question is how we cope with it. If our fear of living meaningless lives arises from our vulnerability and decreasing windows of opportunity, the problem to be solved is how to make wise choices. Flanagan's (7, 8) account deems wise choices as those constituting meaning by opening the windows that in sum attain a lifestory that justifies itself. Nagel (34) argues that although there does not seem to be any cosmological justification that would ensure meaning to us, he also argues that ordinary actions and choices we deem important, valuable and serious, ultimately by other people and even after my death, would be part of a meaningful life. Such a life would be meaningful because I can face death without fear. I can look upon my own life as meaningful even if there isn't meaning in the universe as such. At first sight both Flanagan's and Nagel's positions seem to establish a life's meaning by its consequences for myself, others, and the world. They are externally grounded: My life seems meaningful only if it has positive historical effects. It is not our intention to present Flanagan and Nagel as consequentialists; that would be a misrepresentation. We argue that life is not merely a sequence of events. Life is an ongoing personal interpretation, a narrative self-understanding, a lifestory and eventually a history. It is a positive history if it can be looked back upon without regret, nothing more. On behalf of Flanagan and Nagel, we are not trying to attribute meaning to a life by actual (future) consequences, neither during life nor after death. Nor are we claiming that positive consequences would not be welcome. From the philosophical and existential tradition we are using to discuss meaning and PL, consequences may be better left to other perspectives or instrumental concerns. Existentialism may be criticized for being too individualistic or having an unnuanced view of freedom (see 35 for a discussion). Still, we will continue by holding on to the idea that no matter how much my life is restrained or effected by society and others, my actions and choices and responsibility for them are ultimately mine. It is *my* life that may or may not provide positive historical effects; it is my own judgement that decides what to do or not.

Acknowledging that our opportunities diminish throughout life reveals not only how difficult life can be, but also how significant the choices we make truly are. Knowledge and philosophical contemplation might guide motivation, confidence, and understanding on our physical literacy journey. If our opportunities narrow with every choice, then choosing which movements to carry on through life is among the choices that matter, sometimes even the most. In summary, we can agree with Lucretius on one point: it is not death itself that we should fear. Rather, Sartre (16) before Nagel, and Nietzsche before Sartre all highlighted both the importance and feeling of doom connected to human choice as a way of establishing meaning. How do I make significant decisions? It is a burden, the greatest weight.

### Nietzsche, the greatest weight and the ultimate confirmation and seal

We will now examine Nietzsche's idea of the *eternal recurrence* as a hypothetical thought experiment (36, p. 150) and a potential tool for existential choice. If successful, this may enhance knowledge, confidence and motivation related to movement and activity, thereby possibly fostering lifelong joy, a positive lifestory and a sense of meaning. Nietzsche, together with other philosophers such as Kierkegaard, Heidegger, and Sartre, initiated an existentialist movement in philosophy that came to shape intellectual thought well into the twentieth century. According to Nietzsche, human beings must seek values and meaning elsewhere than in religion and science. We must thus be our own authorities and build our identity and sense of meaning on a different foundation. Nearly 130 years after Nietzsche's death, life in our post- or late modern world may not be all that different. We still require something to help us cope with the meaninglessness of the universe. Here, we can only give a short introduction to Nietzsche. We will elaborate on the idea of the eternal recurrence and the most important aspect of it: What we shall refer to here as Nietzsche's hypothetical existentialism.

*The Gay Science* (19, GS 341) contains what is probably the most famous presentation of the eternal recurrence. Nietzsche asks us to imagine a demon saying to you: “This life as you now live it and have lived it, you will have to live once more and innumerable times more; and there will be nothing new in it, but every pain and every joy and every thought and every sigh and everything utterly small or great in your life will have to return to you, all in the same succession and sequence…(…)Would you not throw yourself down and gnash your teeth and curse the demon who spoke thus? Or have you ever experienced a tremendous moment when you would have answered him: “You are a god and never have I heard anything more divine.” …(…)The question in each and every thing, “Do you desire this once more and innumerable times more?” would lie upon your actions as the greatest weight.”

According to Nehamas (36, p. 142), the eternal recurrence is standardly interpreted as a cosmological hypothesis (e.g., 37, p. 326). As such, the hypothesis claims that everything that has happened, is happening and will happen has already happened before and will do so again in cycles. Events will inevitably recur in the exact same order, thereby being qualitatively identical. There is no variation, and no interaction between these cycles, so it also makes sense to describe all of them as just one. If there is nothing you can do to interact with or change any former, future or current event, this is also to say that there is no difference between a life repeating eternally and just one life lived. It is also to highlight the importance of this one and only life, since it is identical to an eternally recurring life. Nehamas (36, p. 160) interprets Nietzsche as to suggest that all events imply both those events before and those after. As such, if one moment repeated itself then all others must also repeat themselves. In Zarathustra's words: “And are not all things knotted together so firmly that this moment draws after it all that is to come?” (38, Z, III, The vision and the enigma). The eternal recurrence may be interpreted as a hypothesis of holism, in the everyday sense of the word; an individual's life and journey are totally interwoven and constituted in every moment. We will keep this outline of the eternal recurrence, but not as cosmology. We do not believe Nietzsche meant the eternal recurrence is a description of how the universe works, and for our project it makes no difference whether it does or not. It *may* be seen as a thought experiment, and for our purpose one's attitude towards the demon's question is what matters.

In GS 341 then, Nietzsche seems mostly interested in the attitude one must put forward to not let the demon's question crush you. For the individual, Nehamas (36, p. 153) claims, the eternal recurrence helps me understand that *if* my life were to recur, then it could recur only in identical fashion. The demon cannot offer us anything other than *this* life – an alternative would not only be another life, but an entirely different world. This is the reason, insists Nehamas, that there are only two responses to the demon's question. If we curse the demon, we do not confirm our life – we have not lived a meaningful life. On the contrary, if we have experienced moments that made us desire the exact same life over and over again, then we have found meaning, even if by the looks of it - only for a moment. A shrug is not an alternative: This is my life and world we are dealing with - the psychological and existential implications are huge. One implication is that accepting a particular moment is accepting everything else. According to Nietzsche (38, Z, IV), all moments are interwoven with all other events and moments in our lives. The idea is that all our actions and choices make up our lives in the sense that meaningfulness in moment M2 is interwoven with pain/sorrow in moment M1. When at moment M3 I look back upon M2 I will understand that also M1 is a meaningful moment because M2 is connected to and interwoven with M1. By affirming a particular moment as meaningful, I have also affirmed my entire life as meaningful. Accepting a current moment can then re-interpret the past. This moment, now, is such as it is in relation to everything in my life. What I earlier took to be bad may come to be seen as an important experience for my current moment of appreciation. This moment of joy simply might not occur without all the anger and sorrow I have earlier experienced. That is comforting in a sense. It makes every moment count; any moment might become meaningful. In the present moment, every dust and every sigh may reinterpret the past, which again is part of the future. Such an interpretation is also a tough pill to swallow. On the one hand, it seems difficult to re-evaluate at moment E3 an obviously bad event E1 as good because E1 also led to something positive E2. On the other hand, it does not seem Nietzsche (nor Nehamas) argues for a theory of backward causation, where a later event can causally influence an earlier (39). In the latter case, Nietzsche is arguably notoriously skeptical to cause-event relations at all (40, BGE 14, 41, GM 13. See also 42, 43 for discussions on Nietzsche and causality). The former presents a difficult case: Let's say a friend dies in a parachute accident. This incident does not necessarily *cause* me to quit parachuting but is most likely part of me quitting. I then pick up playing chess, which gives me immense joy. Does this mean that when I look back upon my friend's death it is now seen as a good thing since it is connected to my appreciation of chess? Following our presentation of Nietzsche, we may feel inclined to say yes. Reginster (44, pp. 214–217) argues that Nehamas goes too far in claiming that affirming life requires affirming everything within it, since there are clearly events over which a person has no control. In contrast to Nehamas's normative (or even radical) contextualism, Reginster proposes a more moderate version, according to which one aspect of a person's life depends only on *some* other aspects of that life. We may say that to affirm my friend's death as something connected to and intertwined with something new and enjoyable is not to say that my friend's death was good. Instead, we may come to see that loving chess, or any new activity, may call for celebration no matter how bad or evil the circumstances are. *That* may be affirming and reassuring to us (see 45, pp. 245–286 for a discussion of the eternal recurrence, affirmation and a critique of Nehamas). Without going into a lengthy discussion, our interpretation of Nehamas and Nietzsche bears resemblance to Flanagan's perspectives on self-understanding as a narrative and lifestory. Even though Nietzsche explicitly refers to moments while Flanagan and Nagel apparently are more occupied with a lifespan, they all see life as a constant interpretive process. We are not advancing a causal theory regarding any of them. We have argued, however, that the past, present, and future are intertwined with who we are and who we might become. It is perhaps too much though, to argue that a narrative self-understanding, or Nehamas’ interpretation of Nietzsche, leaves us with a theory of indetermination. The question of free will and determinism always lurks below the surface, if committed to physical monism. Here, we can approach such problems only like a swift bath in cold waters, to rephrase Nietzsche (19, GS 381). A weaker claim than a theory of indeterminism is sticking to a theory of interpretation. Nietzsche (19, GS 374) claims that we cannot reject the possibility of infinite interpretations. Combined with what we have called holism in the everyday tongue, the interpretation and narrative of our self-understanding may continually change. Hence, our lives are not determined in the sense that they are unchangeable (see also 44, p. 214).

On our physical literacy journey, our choices of physical activities and our reasons for choosing them become important. Knowledge and understanding will help us choose, maintain, and develop our physical competence, self-confidence, and our identity and lifestory. Our choices, actions and movements are important not only due to our fragility as humans, but also because we spend large parts of our lives partaking in activities that often include others and build valuable relationships. Both moments of struggle and moments of joy are embedded and intertwined in our physical literacy journey. Choosing actions that may fill life with meaningful moments, and what we have loosely called a positive historical effect might be the way to create meaning in a meaningless physical universe. It is also a heavy burden, the greatest weight we can put upon ourselves. But by carrying this weight, by not being a passive but an *active* nihilist as Nietzsche (46, WP 22) calls it, we move and affirm who we are. We *might* overcome the loneliness and void of the universe and our life if we make this struggle part of our self-expression, part of our identity. This is not an end, but an ongoing process. It is both the philosophy and the movement of the physical literacy journey.

### A journey becoming

Throughout this article, we have sought to tie together several philosophical themes surrounding PL and meaning. We have argued that meaning on our physical literacy journey is established through choices about why, how and which movement activities we commit to. Our presentation of the thought experiment in the eternal recurrence has been framed as a hypothetical question: if you think about life as repeating itself eternally, what would that imply for the choices you make and the way you live your life? This is partly the same question as what makes life worth living, leading up to Owen Flanagan's answer: self-expression and identity. As we have elaborated upon, not only great achievements express who we are and thus create meaning in life. Meaning can also be established by what we usually find to be the small things. Physical activity might be considered such a small thing. Nevertheless, physical activity expresses myself; showing who I am by becoming a moving body. Accepting physical monism is accepting that I express myself bodily and thus movement is a privileged, if not necessarily the supreme, way to do it.

Our focus has been philosophical rather than didactical, but a brief word on implications for physical education (PE) and physical education teacher education (PETE) is warranted. The demon's hypothetical question in Nietzsche's aphorism can be presented and discussed according to age with PETE students and PE pupils. We are not proposing that the aphorism be taught as some kind of doctrine, but PETE should prepare future teachers to reflect on existential questions just as much as on organizing and designing activities. PE as a subject is often justified by what it supposedly produces in terms of health, activity levels, and the prevention of later inactivity. A shift away from instrumental justifications and more towards existential dimensions might even strengthen PE's place at both school and university levels.

Existentially put, choosing to be physically active resembles life in its most basic dimension: Why do we live? I don't know, I just have to. But if I can withstand the dreadfulness of looking into the abyss of a meaningless universe, there might be a tremendous moment. I have experienced such a moment, once or twice. What if? A perfect move, a perfect place, such a moment. Again. And again. If physical activity is chosen in a knowledgeable, confident, philosophical, and meaningful way, it might not only be the summit and/or fundament of physical literacy, but even a vital part of *life* literacy. If so, I am always on a journey towards becoming a physically literate person. Or rather: I move, therefore I am.
